# Compliance with antibiotic prophylaxis guidelines in caesarean delivery: a retrospective, drug utilization study (indication-prescription type) at an Ecuadorian hospital

**DOI:** 10.1186/s13756-020-00843-1

**Published:** 2021-01-12

**Authors:** Katherine Romero Viamonte, Adrian Salvent Tames, Rosa Sepúlveda Correa, María Victoria Rojo Manteca, Ana Martín-Suárez

**Affiliations:** 1 Faculty of Health Sciences, Technical University of Ambato, Ambato, Ecuador; 2 Pharmaceutical Sciences Department, University of Salamanca, Salamanca, Spain; 3 Obstetrics and Gynaecology Department, Ambato General Hospital, Ambato, Ecuador; 4 Statistical Department, University of Salamanca, Salamanca, Spain; 5 Provincial Pharmacists Chamber, Ávila, Spain

**Keywords:** Surgical antibiotic prophylaxis, Caesarean section, Surgical site infections, Cost saving, Clinical practice guidelines

## Abstract

**Background:**

Preoperative antibiotic prophylaxis is essential for preventing surgical site infection (SSI). The aim of this study was to evaluate compliance with international and local recommendations in caesarean deliveries carried out at the Obstetrics and Gynaecology Service of the Ambato General Hospital, as well as any related health and economic consequences.

**Methods:**

A retrospective indication-prescription drug utilization study was conducted using data from caesarean deliveries occurred in 2018. A clinical pharmacist assessed guidelines compliance based on the following criteria: administration of antibiotic prophylaxis, antibiotic selection, dose, time of administration and duration. The relationship between the frequency of SSI and other variables, including guideline compliance, was analysed. The cost associated with the antibiotic used was compared with the theoretical cost considering total compliance with recommendations. Descriptive statistics, Odds Ratio and Pearson Chi Square were used for data analysis by IBM SPSS Statistics version 25.

**Results:**

The study included 814 patients with an average age of 30.87 ± 5.50 years old. Among the caesarean sections, 68.67% were emergency interventions; 3.44% lasted longer than four hours and in 0.25% of the deliveries blood loss was greater than 1.5 L. Only 69.90% of patients received preoperative antibiotic prophylaxis; however, 100% received postoperative antibiotic treatment despite disagreement with guideline recommendations (duration: 6.75 ± 1.39 days). The use of antibiotic prophylaxis was more frequent in scheduled than in emergency caesarean sections (OR = 2.79, P = 0.000). Nevertheless, the timing of administration, antibiotic selection and dose were more closely adhered to guideline recommendations. The incidence of surgical site infection was 1.35%, but tended to increase in patients who had not received preoperative antibiotic prophylaxis (OR = 1.33, P = 0.649). Also, a significant relationship was found between SSI and patient age (χ^2^ = 8.08, P = 0.036). The mean expenditure on antibiotics per patient was 5.7 times greater than that the cost derived from compliance with international recommendations.

**Conclusions:**

Surgical antibiotic prophylaxis compliance was far below guideline recommendations, especially with respect to implementation and duration. This not only poses a risk to patients but leads to unnecessary expenditure on medicines. Therefore, this justifies the need for educational interventions and the implementation of institutional protocols involving pharmacists.

## Introduction

Caesarean section is one of the most frequent obstetric surgeries in the world and its use has increased exponentially in recent years [1]; it allows the life of the mother and/or child to be saved in certain situations, but it is not without risk [2].

Complications of caesarean delivery include surgical site infections (SSIs), which are among the leading causes of maternal death [3]. Morbidity from infection has been reported to be about eight times higher after caesarean delivery compared to vaginal delivery, with an SSI rate between 3 and 15% [4], although according to other studies it may be as high as 25% [4, 5].

Appropriate preoperative antibiotic prophylaxis (PAP), defined by the World Health Organization (WHO) as "administering an effective antimicrobial agent prior to exposure to contamination during surgery", is necessary to prevent SSI [5].

Although multiple studies have shown the importance of the PAP in minimising risks and optimising available institutional resources, recommendations are often not followed  [6–8].

In Ecuador, there is a Clinical Practice Guide for Caesarean Delivery Care (CPG—Ecuador)  [9] that addresses PAP but does not establish specific protocols for antibiotic use.

Based on the above, the objective of this study is to assess compliance with international and local recommendations on PAP in caesarean delivery at the Obstetrics and Gynaecology Service of Ambato General Hospital as well as its economic impact.

## Methods

A retrospective indication-prescription drug utilization study was conducted. All women who, irrespective of the cause, underwent a caesarean birth in the Obstetrics and Gynaecology Service at the Ambato General Hospital in the year 2018 were considered as the study population. However, some of them were excluded: patients lacking an agreed diagnosis or personal data at the time of the review; patients with Premature Rupture of Membranes (PROM) remote from term (from 24 to 34.6 weeks), as they required another type of prophylaxis not studied in the present study [10]; and patients undergoing antibiotic treatment for an active clinical infection. Thus, the final sample size was 814 patients. The identification of the diagnosis was made in accordance with the International Classification of Diseases, 10th edition (ICD-10).

### Information collection

The information of the patients was obtained from the review of medical records through the file of hospital discharge reports of the Obstetrics and Gynaecology Service from the Medical Information System (MIS/AS400). It included the following data: age, origin, dates of admission and hospital discharge, date and type of surgery (scheduled or emergency), estimated blood loss and SSI.

The following aspects were recorded in relation to PAP: whether or not it was applied, antibiotics used, dose, timing of administration and duration.

### PAP compliance assessment

A clinical pharmacist retrospectively assessed whether preoperative prophylaxis had been adequately used, considering the directives stated in the CPG—Ecuador [9] and the American Society of Health-System Pharmacists (ASHP) Guideline  [11]. The following criteria were considered:

#### PAP indication

1. Appropriate indication: administering preoperative prophylactic antibiotic treatment before making the skin incision, unless the patient has an active infection for which she is already receiving antibiotic treatment  [9, 11].
2. Inappropriate indication: when the above is not complied with.

#### Selection criteria for antibiotics

1. Appropriate selection of antibiotic: using first generation cephalosporins (cefazolin) or joint treatment of gentamicin and clindamycin in patients allergic to beta-lactams  [11].
2. Inappropriate antibiotic selection: using any antibiotic other than those mentioned above. Also included in this category is the use of combination therapies by two or more antibiotics with similar activity spectra for which there is no evidence demonstrating synergistic activity.

#### Antibiotic dose

1. Appropriate dose: cefazolin IV at 2 g (3 g for patients weighing more than 120 kg), clindamycin IV at 900 mg and gentamicin IV at 5 mg/kg  [11].
2. Inappropriate dose: using antibiotics at doses other than those referred to above.

#### Time of antibiotic prophylaxis

1. Appropriate timing of administration: receiving the antibiotic intravenously during the 60 min prior to the surgical incision. In the case of emergency surgery, administration up to the point of incision was considered appropriate  [9, 11].
2. Inappropriate administration time: receiving an intravenous antibiotic at any other time before or after the incision.

#### Duration of antibiotic prophylaxis

1. Appropriate duration: administering a single dose of intravenous antibiotic or, in the case of operations longer than 4 h or blood loss greater than 1.5 L, prolonging treatment by no more than 24 h after surgery  [11].
2. Inappropriate duration: prolonging the administration of antibiotics for more than 24 h after the end of surgery.

### Cost analysis

For the cost analysis, only the expenditure associated with the use of antibiotics was taken into account. The unit price of each of the drugs used for PAP was defined based on the information provided by the Pharmacy Department of the institution under study.

The cost of treatment was obtained from the dosage schedule used in each of the patients studied. On the other hand, the ideal cost was calculated considering the compliance with the PAP as established in the reference guidelines and the PAP cost/patient ratio.

The difference between the real cost of the treatments administered and the ideal cost obtained in the research indicates the savings that would have been made by optimising compliance with the recommendations.

### Statistical analysis

IBM SPSS Statistics version 25.0 was used for data analysis. Categorical/Binary variables were presented as frequency and percentage while continuous variables were reported as mean ± standard deviation. Association between categorical variables was determined using Pearson Chi Square; the Odds Ratio was used to report the statistical association between binary variables. P value < 0.05 was considered as statistically significant.

### Ethical considerations

Prior to the start of the study, the protocol was reviewed by the relevant authorities at Ambato General Hospital, who approved the study as meeting the ethical requirements of the institution.

Data analysis was performed in a coded file to preserve patient anonymity.

## Results

Table 1 shows the socio-demographic and clinical characteristics of the 814 patients included in the study. The mean age was 30.87 ± 5.50 years, with the majority of patients being between 20–34 years of age. Most of the patients were from urban areas and underwent emergency surgery. The average duration of surgery was 173.54 ± 46.66 min and only 3.44% of the interventions exceeded four hours. In 0.25% of cases there were blood losses of more than 1.5 L, and 1.35% (11 patients) had SSI. The mean hospital stay was 3.86 ± 2.59 days.

**Table 1:** Sociodemographic and clinical characteristics of the patients included in the study (n = 814)

Variables	N (%)
*Age (years)*	
16–19	16 (1.97)
20–34	584 (71.74)
≥ 35	214 (26.29)
*Place of origin*	
Urban	544 (66.83)
Rural	270 (33.17)
*Type of surgery*	
Scheduled	255 (31.33)
Emergency	559 (68.67)
*Duration of surgery (hours)*	
< 4	786 (96.56)
≥ 4	28 (3.44)
*Blood loss (litres)*	
< 1.5	812 (99.75)
≥ 1.5	2 (0.25)
*Surgical wound infection*	
Yes	11 (1.35)
No	803 (98.65)

Table 2 summarizes the patterns of antibiotic used in the patients studied. 69.90% were administered PAP using four different therapeutic schemes. The predominant treatment was cefazolin 2 g IV (92.44%); clindamycin was reserved for patients allergic to penicillin (1.76%), but in no case was it combined with aminoglycosides, as indicated by ASHP.

**Table 2:** Use of antibiotics in study patients (n = 814)

***Preoperative administration***			
Yes 569 (69.90)*	No 245 (30.10)		
*Administration time*	*Antibiotic and administered doses*		
Within 60 minutes before the incision At the time of incision	212 (37.26) 357 (62.74)	Ampicillin/sulbactam 1.5 g IV Cefazolin 1 g IV Cefazolin 2 g IV Clindamycin 600 mg IV	1 (0.18) 32 (5.62) 526 (92.44) 10 (1.76)
***Postoperative administration***			
814 (100)			
***Duration of antibiotic treatment***			
Single dose	–		
24 h	–		
> 24 h	814 (100)		

^*^Number of patients (%)

All patients received postoperative antibiotics, with a mean duration of 6.75 ± 1.39 days. Thus, 88.67% of them were prescribed parenteral antibiotics during the 24 to 72 h after surgery following 25 different therapeutic schemes, and in the rest the oral route was used. The most prescribed intravenous antibiotics were cefazolin, clindamycin and ampicillin/sulbactam. After the first 24–72 h, 95.75% of the patients continued the treatment orally in the form of 21 therapeutic schemes; in nine patients, eight schemes combining oral and parenteral medicines were used. The most prescribed oral antibiotics were cephalexin, clindamycin and amoxicillin/clavulanic acid. Upon discharge from the hospital, the patients received, free of charge, the medicines to complete the treatment.

Table 3 summarises compliance with the recommendations of the selected reference guidelines based on the criteria set out above. Regarding the selection of the antibiotic and dose used, it was not possible to use the CPG-Ecuador because it does not refer to specific drugs or doses.

**Table 3:** Compliance with the recommendations of the reference guidelines for preoperative antibiotic prophylaxis

Assessment criteria	CPG- Ecuador N (%)	ASHP N (%)
Use of PAP	569 (69.90)*	569 (69.90)*
Appropriate selection of the antibiotic	−	558 (98.07)**
Appropriate dose of the antibiotic	−	526 (92.44)**
Appropriate timing of administration	569 (100)**	569 (100)**
Appropriate duration of administration	0	0

*CPG-Ecuador* Clinical Practice Guidelines for caesarean delivery care in Ecuador, *ASHP* American Society of Health-System Pharmacists guideline, *PAP* preoperative antibiotic prophylaxis

*Percentage of the total population included in the study (n = 814); **Percentage of the population that received PAP (n = 569)

With regard to costs associated with treatment, which are reflected in Table 4, a total of USD 2 743.36 was spent on antibiotic management of patients undergoing caesarean delivery during the study period.

**Table 4:** Costs associated with the use of antibiotics in the patients studied

PAP assessment criteria		Patients (N)	Pharmaceutical form (Unit)		Cost of treatment (USD)		Global cost
			Capsule	Vial	Capsule	Vial	USD (%)
Preoperative administration							
Appropriate antibiotic selection		558	–	1084	–	262.11	262.11 (9.55)
Inappropriate antibiotic selection		11	–	11	–	18.44	18.44 (0.67)
No use of antibiotics		245	–	–	–	–	–
Postoperative administration Inappropriate							
Unsupported indication		814	23,055	4269	1033.04	1429.77	2462.81 (89.78)
Total		814	23,055	5364	1033.04	1710.32	2743.36 (100)

The ideal cost of the PAP, assuming that the recommendations of the ASHP had been followed in all the patients studied, would be USD 476.52. Therefore, compliance would result in savings of USD 2 266,83 (82,63% of the amount spent). The ideal PAP/patient ratio is 0.59 cents, but the actual average expenditure per patient was USD 3.37, almost six times more than would have been necessary if the protocols had been followed.

## Discussion

Compliance with PAP is an important factor in reducing the incidence of SSI and avoiding the costs associated with it  [12]. As in other non-infected surgical acts, antibiotic prophylaxis is recommended for all caesarean deliveries unless the patient is already receiving an antibiotic regimen for another existing infectious entity  [9, 11, 13–16]. However, in our study only 569 patients (69.90%) received PAP.

PAP was administered to a higher percentage of patients in scheduled caesarean Sects. (83.14%) than in emergency caesarean Sects. (63.83%) (OR = 2.79, P = 0.000). No data have been found in the literature to corroborate this observation.

Furthermore, all women, including those who did not receive PAP, received post-surgical antibiotic treatment, something which is non-compliant with the guidelines recommendations. This is the most relevant result of the study and is consistent with that obtained by Saied et al. in Egypt  [17]. Other studies have also shown the inappropriate duration of PAP and the use of different therapeutic schemes that are poorly described and unnecessary  [7, 8, 15, 18, 19]. Patients who suffered a loss of blood greater than 1.5 L or who had prolonged surgery (more than 4 h), received prophylaxis for approximately 7 days, just like the rest of patients, which is in contradiction to what was stated in the literature [11, 14, 15].

Several studies have shown that there is no significant difference in the incidence of postpartum infectious morbidity between the use of single and multiple doses of PAP  [16, 20–23]. The excessive use of antibiotics, could favour the emergence of microbial resistances, increase the risk of adverse reactions and generate unnecessary costs to the institution. The above findings highlight the need for strategies to increase raise the level of practitioner adherence to PAP-use recommendations.

On the other hand,other variables studied showed compliance with the reference guidelines recommendations such as: the timing of administration, the selection of antibiotic and the dose administered (Table 3).

New research continues to recommend that PAP be administered within 60 min before the incision and, in the case of emergency surgery, as soon as possible after the incision,  [8, 9, 11, 16, 17]. In the present study, the timing of PAP administration in all patients was considered appropriate, in contrast to the 80% non-compliance reported by Abubakar et al. [7].

Antibiotic selection was consistent with ASHP in 98.07% of patients receiving PAP and the correct drug dose was administered to 94.27%; these results were similar to those obtained by Abdel Jalil et al.  [15]. CPG-Ecuador does not recommend specific antibiotics, which could favour the use of a wide range of therapeutic schemes, as observed in this study.

There is widespread agreement on the use of first generation cephalosporins (cefazolin), or a combination of aminoglycosides and clindamycin for patients with a history of severe reaction to cephalosporins, to avoid SSI in most surgical procedures  [11, 24, 25]. However, there are other proposals in terms of antibiotic selection and dosage for PAP in caesarean sections  [16, 26, 27]. The difference could be justified by the characteristics of the circulating germs, the prescribing habits in each institution or the non-existence, inadequate design or non-compliance of clinical guidelines intended for this purpose.

The incidence of SSI in this study (1.35%) is lower than in other similar studies, where it reaches up to 40%  [19, 28–31]. In patients who were not given PAP, the incidence of SSIs was higher than in those who received pre-surgical antibiotics (1.63% *vs* 1.23%). At the sample level it is observed that the ratio between presence and absence of SSIs is 1.33 times higher in subjects without PAP as compared to subjects with PAP (OR = 1.33; P = 0.649). This difference increases (1.14 vs 1.74) if we compare patients who received the correct antibiotic at the appropriate dose (6 cases in 526) with those who did not receive PAP or who did not receive an appropriate choice of antibiotic and/or dose administered (5 cases in 288) (OR = 1.53, P = 0.485). These results may indicate a tendency to decrease the development of SSI when there is greater compliance with the guidelines' recommendations. It should be taken into account, however, that in all patients a post-surgical antibiotic was used for several days, which makes it difficult to statistically demonstrate the benefits of PAP on this variable.

It has also been observed that the incidence of SSIs increases with the age of the patients (16–19: 0%; 20–34: 0.7%; 35 or older: 3.3%). Thus, a significant relationship was found between these variables (χ^2^ = 8.08, P = 0.036), which is in line with similar data reported in other studies  [31–33]. In contrast, no association was found between patient age and PAP administration (χ^2^ = 1.59, P = 0.44).

90.45% of the expenditure on antibiotics was associated with their inappropriate use, mainly due to their administration after surgery. The ideal PAP cost per patient was 0.59 USD; however, the actual average expenditure per patient was 3.37 USD, i.e. almost six times more (5.7) than needed (Table 4). Although the figures may seem small, the institution has limited capacity for acquiring resources that are indispensable in the health care of other patients.

Unfortunately, little research addresses the issue of costs of noncompliance with the PAP in caesarean sections. Instead, studies generally address the average cost of a patient receiving PAP and how cost-effective it is compared to a patient who does not receive it  [32–34]. Jansson et al. found 99% savings from compliance with the PAP,  [35] which is even higher than what was found in the present research.

The participation of the pharmacist within the health care team translates into a decrease in unnecessary costs, an increase in the quality of care and an improvement in the patient's quality of life. These results have led to the acceptance of this professional by other members of the health team  [36–41]. To date, there is no clinical pharmacist at the target institution involved in the design, implementation and review of therapeutic protocols, including PAP.

The fact that the study conducted was retrospective is one of its limitations, as it made it difficult to analyse variables that would allow the risks associated with inappropriate duration of antibiotic use to be assessed.

## Conclusions

The study shows a low compliance with the recommendations of the reference guidelines and the general literature in terms of application, selection and duration of antibiotic prophylaxis, which poses a risk to the health of patients and unnecessary expenses for the institution. In addition, it could have a negative effect on public health through increased bacterial resistance.

The analysis of the PAP in the study sample allowed the detection of weaknesses in CPG-Ecuador, which will serve as a basis for the design of institutional preoperative antibiotic prophylaxis policies that clearly detail which antibiotics to use and at what dose, time and duration.

We believe that the intervention of the pharmacist in the process of design, implementation and assessment of PAP protocols could improve the use of antibiotics and their cost effectiveness.

## Data Availability

The data that support the findings of this study are available from the Obstetrics and Gynaecology Service of the Ambato General Hospital, but restrictions apply to their availability, so they are not publicly available. However, the data may be obtained from the authors upon reasonable request and with the permission of the Obstetrics and Gynaecology Service of Ambato General Hospital.

## Abbreviations

SSIs: Surgical site infections
USD: United States Dollar
PAP: Preoperative antibiotic prophylaxis
WHO: World Health Organization
CPG-Ecuador: Clinical Practice Guidelines for caesarean delivery care in Ecuador
ICD-10: International Classification of Diseases, 10th edition
PROM: Premature rupture of membranes
MIS/AS400: Medical Information System
ASHP: American Society of Health-System Pharmacists guidelines
